# Supplementary material for: Welfare Implications of Low-Dose Atipamezole Reversal of Tiletamine/Zolazepam/Xylazine Anaesthesia in Pigs
Source: Animals (Basel). 2025 Jan 17;15(2):258. doi: 10.3390/ani15020258 (PMC11758624; doi:10.3390/ani15020258)
Supplement: Supplementary file 1 [file animals-15-00258-s001.zip › animals-3404070-supplementary.pdf]

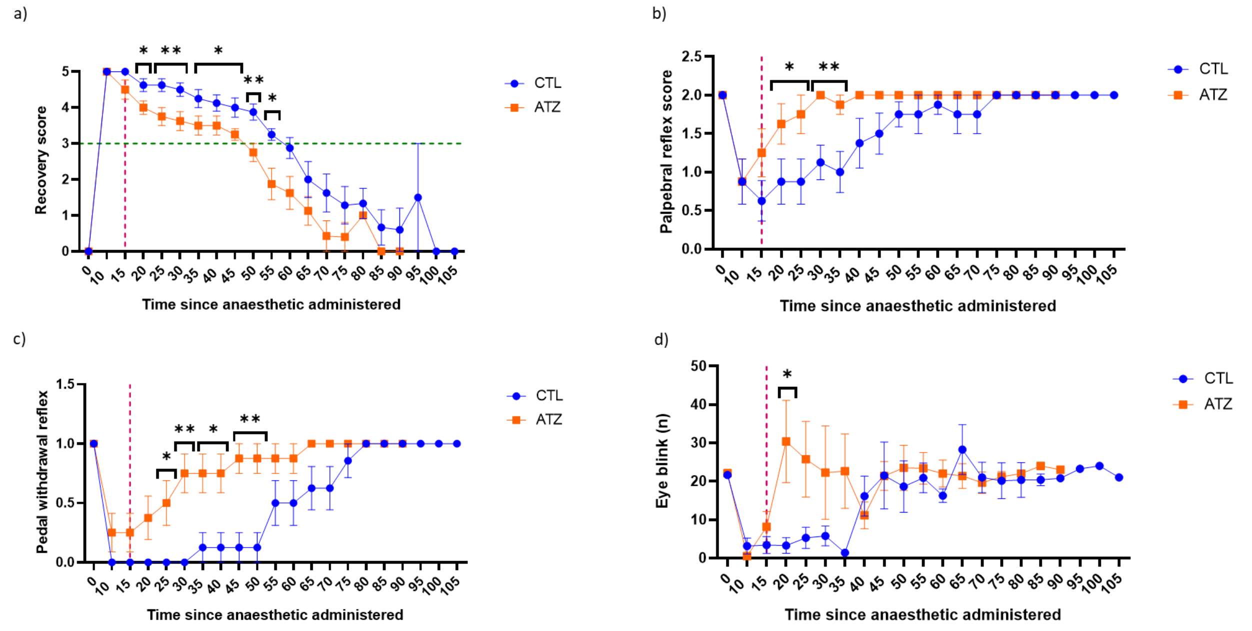

Supplementary figure 1: Pigs administered a low dose of atipamezole recover more rapidly from anaesthesia. Prior to and following the administration of anaesthetic we measured the recovery score (a), palpebral reflex (b), pedal withdrawal (c) and eye blink frequency (d) at 5-minute intervals. Orange line represents atipamezole treated compared to blue control. Green line represents return to connected consciousness (a). Pink line represents time of atipamezole administration for ATZ group. Palpebral reflex was scored as absent (0), slight (1) or fully present (2) (b). Pedal withdrawal reflex was scored as absent (0) or present (1) (c). Error bars represent standard error of the mean. Statistical significance was calculated using multiple paired t-tests with the following degrees of significance: *= p<0.05, **= p<0.01.

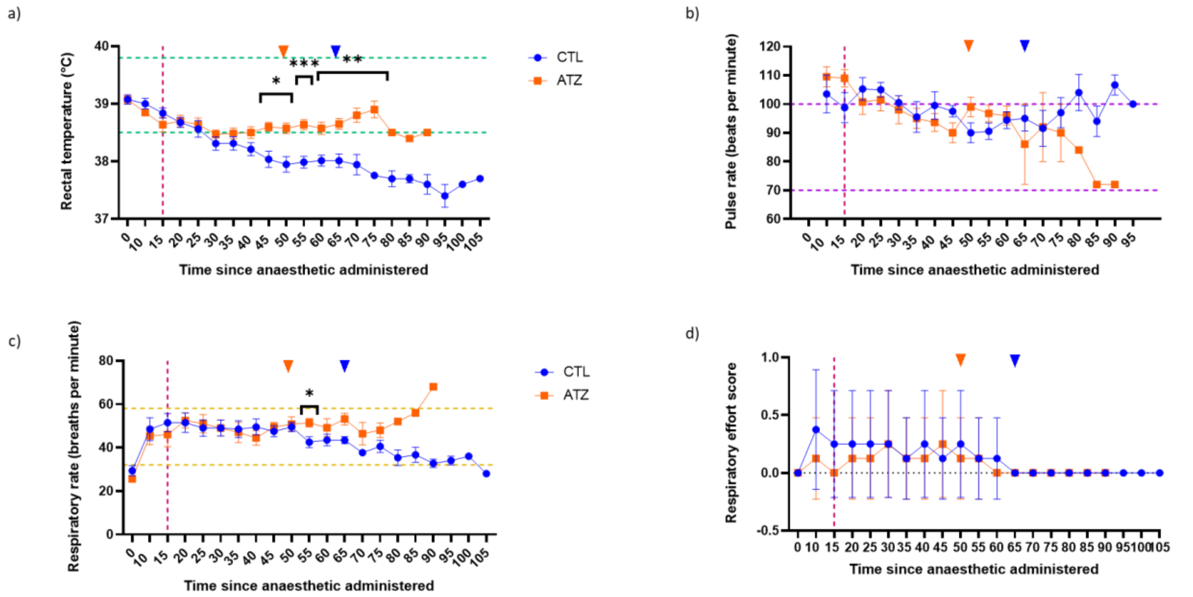

Supplementary figure 2: Low dose atipamezole reduces some, but not all, physiological impacts of anaesthesia in pigs. Prior to and following the administration of atipamezole we measured rectal temperature (a), pulse rate (b), respiratory rate (c) and respiratory effort (dd) at 5-minute intervals. Arrows indicate the return of consciousness for ATZ (orange) and CTL (blue) pigs. Pink vertical line represents the time of atipamezole administration. Green lines represents normal rectal temperature range for growing pigs (a). Purple lines represent the upper and lower normal heart rates for growing pigs (b). Yellow lines represent the upper and lower normal respiratory rates for growing pigs (c). Respiratory effort was scored as normal (0) or increased (1) (d). Error bars represent standard error of the mean. Statistical significance was calculated using multiple paired t-tests with the following degrees of significance: *= p<0.05, **= p <0.01, ***= p<0.001.
